# Korean Hospital Nurses’ Experiences with COVID-19: A Meta-Synthesis of Qualitative Findings

**DOI:** 10.3390/healthcare12090903

**Published:** 2024-04-26

**Authors:** Suk-Jung Han, Hee-Jung Hong, Bok-Soon Shin

**Affiliations:** 1 College of Nursing, Sahmyook University, Seoul 01795, Republic of Korea; hansj@syu.ac.kr; 2 Department of Nursing, Severance Hospital, Seoul 03722, Republic of Korea; 3 College of Nursing, Inha University, Incheon 22212, Republic of Korea; shinboksoon@inha.ac.kr

**Keywords:** nurses, COVID-19, hospitals, qualitative research

## Abstract

This study aims to provide a meta-synthesis of qualitative studies examining the perceptions and experiences of nurses who cared for patients in dedicated COVID-19 hospitals in South Korea. We searched key health databases (RISS, KISS, KMbase, NDSL, KoreaMed, DBpia, PubMed, CINAHL, and Cochrane) from September to November 2023. We reviewed and analyzed articles using a thematic synthesis approach. The quality of the studies was ascertained using the Critical Appraisal Skills Program checklist for qualitative research. Ultimately, 13 studies involving 219 nurses were included in the final review. Six major themes and thirteen subthemes emerged. During the unexpected COVID-19 pandemic, nurses were able to overcome difficult situations through their interactions with patients, sense of a professional mission, and commitment to nursing. Most importantly, they persevered through their collaboration and closeness with fellow nurses, despite confusion about their professional identity, the ethical dilemmas they faced in patient care, and the conflicting attitudes of their social support system. To prepare for future infectious disease outbreaks, a multifaceted support system should be established to enable nurses to have positive interactions with their families, colleagues, and patients, which have become central to their resilience.

## 1. Introduction

The 2019 Coronavirus disease (COVID-19) is an acute respiratory syndrome first reported in Wuhan, Hubei Province, China, on 8 December 2019. The World Health Organization (WHO) declared a global pandemic on 11 March 2020, which corresponds to the highest risk level among the WHO’s pandemic alert levels [1].

The WHO announced that it would declare COVID-19 a Public Health Emergency of International Concern (PHEIC) on 5 May 2023. This officially ended the PHEIC, which was in place for three years and four months since declared on 30 January 2020. According to the WHO’s Coronavirus Dashboard, as of 10 May 2023, the cumulative number of COVID-19 cases worldwide is close to 766 million. Deaths from the disease are reported to be close to 7 million [2].

A survey of healthcare workers infected with COVID-19 in Wuhan, China, found that nurses, who spend the most time with patients on the frontline, accounted for 52.1% of infected healthcare workers, compared to 33.6% of doctors and 14.3% of other healthcare workers [3]. Nurses are not only at risk of infection from caring for patients with infectious diseases, but also experience more mental and physical health issues than other healthcare workers [3,4,5,6,7].

In South Korea, the first case was reported on 20 January 2020, and as of 31 August 2023, the total cumulative number of cases was 34,572,554 (including 79,925 international arrivals) and 35,605 deaths [8].

COVID-19 is highly contagious and asymptomatic in its early stages. Thus, nurses were working under conditions of high anxiety and with a fear of infection. There were also conflicts and confusion about the stress of the pandemic, donning and doffing Level D protective clothing, and nursing roles [9]. These experiences may vary depending on the social, cultural, and situational context of the country, as well as COVID-19-related health policies, nurses’ social status, societal perceptions of nurses, and healthcare resources [10]. Certainly, the success of an infectious disease response depends on strategies to manage the human factors of the healthcare workforce, especially those with a direct and critical role in patient care [11]. Therefore, examining the experiences of healthcare workers who transitioned and operationalized hospital functions and systems in a short period is important in shaping policy for future infectious disease responses.

Nurses reported that in the early stages, they overcame these challenges through psychological adjustment, life adjustment, altruistic behavior, team support, and rational thinking. In the later stages, they reported increased affection and gratitude, development of professional responsibility, and opportunities for self-reflection [12]. However, nurses also self-isolated out of fear of infecting others with COVID-19, which led to social isolation, depression, and physical, mental, and social stress [9].

Based on previous experience with infectious disease outbreaks such as the H1N1 influenza in 2009, Middle East respiratory syndrome (MERS) in 2015, and COVID-19 in 2019, humans can predict other emerging infectious disease outbreaks [13]. Therefore, the experience of nurses caring for COVID-19 patients in the context of a protracted pandemic needs to be more closely scrutinized [14].

A meta-analysis encompasses the knowledge that can be accessed by reviewing topics in a research area and the process by which that knowledge can be expanded. They guide new contributions that are important in understanding and advancing a research area [15].

Therefore, this study aims to compare, analyze, interpret, and synthesize the results of qualitative studies on the patient care experiences of hospital nurses in Korea during the COVID-19 pandemic. The purpose is to better understand the context and outcomes of meaningful common experiences and adaptations in nurses’ experiences. Furthermore, this study aims to provide a basis for the development of practical coping strategies for hospital nurses caring for patients with emerging infectious diseases in the future.

## 2. Materials and Methods

This research is a qualitative meta-synthesis study that analyzed and synthesized individual qualitative research results on the experiences of nurses during COVID-19.

The research question explored through each qualitative study is: “What are the experiences of hospital nurses in South Korea during the COVID-19 pandemic?”

This study adhered to the guidelines in “Enhancing Transparency in Reporting the Synthesis of Qualitative Research” (ENTREQ) [16].

### 2.1. Search Strategy and Selection Criteria

This study searched academic databases for qualitative studies on the experiences of Korean nurses during the COVID-19 pandemic published until 2023. All searches took place from the inception, with final searches taking place from September to November 2023.

The six major databases in Korea are the Research Information Sharing Service (RISS), Korean studies Information Service System (KISS), Korean Nursing Database (KMbase), National Digital Science Library (NDSL), KoreaMed, and DataBase Periodical Information Academic (DBpia). The three major international databases are PubMed, Cumulative Index of Nursing and Allied Health Literature (CINAHL), and Cochrane. These nine databases were used in the study.

The search was divided into domestic and international publications. Search expressions were constructed by concatenating keywords and MeSH terms with an “AND”. Repeated searches were conducted using nurse* or nurse (MeSH terms), COVID-19 (MeSH term), experience, and Korea (MeSH term). To meet the needs of different databases, the search strategy was tailored to each. For the international databases, we used only “Korea” as a search filter to ensure a comprehensive search (Appendix A).

### 2.2. Inclusion and Exclusion Criterion

The detailed inclusion and exclusion criteria used to select articles that would be useful as data for the meta-synthesis of nurses’ COVID-19 experiences are as follows.

Inclusion criteria for the study subjects were as follows: (1) The study population was limited to nurses who worked in hospitals in South Korea during the COVID-19 pandemic. (2) Only articles that specified the use of qualitative research, such as phenomenological and content analysis methods, and analyzed the results according to the specified data analysis methods were included. (3) Only articles published in Korean or English were included.

Articles were excluded if they (1) did not present specific qualitative research methods or presented only results without specifying the data collection and analysis process; (2) were not published in a journal, such as a thesis; and (3) were written in a language other than Korean or English.

### 2.3. Study Inclusion

**Figure 1:**
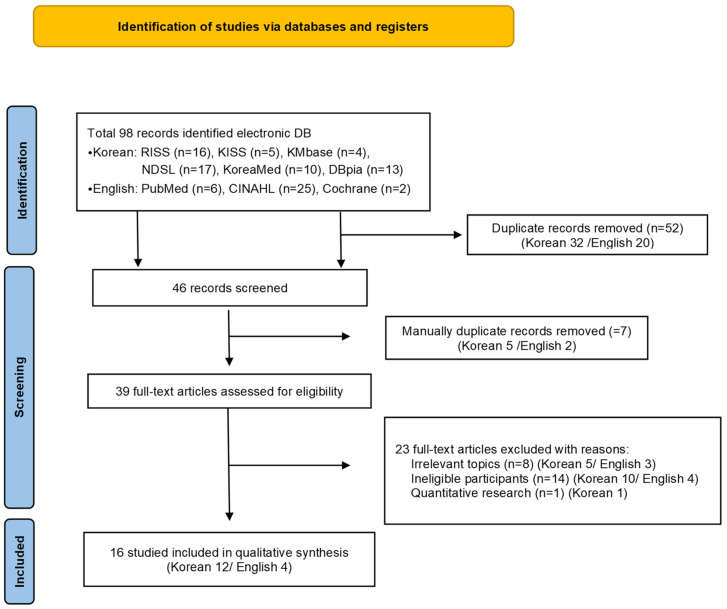
Flowchart of article extraction process.

The screening process adhered to the Preferred Reporting Items for Systematic Reviews (PRISMA) protocol [17]. Figure 1 shows a flowchart of the selection process.

In total, 98 articles were retrieved from the literature search, including 16 from RISS, 5 from KISS, 4 from KMbase, 17 from NDSL, 10 from KoreaMed, 13 from DBpia, 6 from PubMed, 25 from CINAHL, and 2 from Cochrane, and 52 duplicates were removed using EndNote. The titles and abstracts of each study were reviewed. Consequently, an additional 7 duplicates were removed, resulting in 39 primary extracts.

After reading and checking the full text of the 39 extracted articles, we found 8 studies that were not relevant to the topic. These focused on experience with a COVID-19 patient care education program (1), perinatal and neonatal nursing experiences (1), parenting experiences of nurses with elementary school children (1), experiences of hospice palliative care nurses (1), experiences of attending a graduate school for a nurse practitioner program (1), emergency nurse–patient communication (1), art-based narrative research (1), and experiences of wearing personal protective equipment (1). Furthermore, in 14 studies, participants were not nurses who directly cared for COVID-19 patients (ineligible participants). The participants in these studies were nurse managers (1), new nurses (2), nursing home nurses (4), volunteers (1), infection control nurses (1), health center nurses (1), nursing students (3), and airline nurses (1). Thus, 23 articles were removed, including 1 quantitative study. After a secondary screening, 16 studies were selected for the purposes of our study. Of these, 12 articles were written in Korean and 4 in English (Figure 1).

### 2.4. Study Selection

All articles found through the search were sent to Endnote20 (Clarivate Analytics, Philadelphia, PA, USA) and duplicates were removed. Two reviewers (Han, S.-J. and Hong, H.J.) screened the titles and abstracts of the articles against the inclusion and exclusion criteria, and then read the full text of articles to identify those that met these criteria.

Two researchers independently reviewed the data and decided on the selection. In the case of disagreement or uncertainty, the researchers exchanged opinions and reached a consensus, and the reasons for selection or deletion were recorded.

We removed some studies from the list because they were not relevant to hospital nurses, such as those about the experiences of nurses working for airlines, those that focused solely on the experience of wearing PPE, the experiences of nurse managers in nursing homes, the experiences of nurses working in drive-through screening centers, and other quantitative studies.

### 2.5. Quality Assessment

Three reviewers (Han, S-J., Hong, H.-J. and Shin, B.-S.) critically appraised the quality of the articles included in this study using the Critical Appraisals Skills Programme (CASP) [18]. Any disagreements about the quality assessment were discussed until a consensus was reached.

In this study, the 16 included articles were cross-appraised by three researchers using the CASP checklist items to assess their quality. 

The quality assessment consists of 10 questions dealing with the following aspects: “Clarity of statements”, “appropriateness of qualitative research methods”, “appropriateness of research design”, “appropriateness of data collection”, “relationship between researcher and participants”, “consideration of ethical issues”, “rigor of data analysis”, “clarity of results statements”, and “value of the study”. Each representative question has a detailed description and can be answered with yes/no or state that no answer was provided.

The quality assessment is presented in Table 1.

The quality assessment resulted in 1 yes/no or no answer in 3 of the 13 studies. When researchers had different ratings for the same study, consensus was reached through discussion. While assessing the quality of individual articles for a synthesis is an important step, there is no consensus on its benefits. It has also been recommended that the results of the assessment should not be used as a criterion for deciding whether to include an article in the analysis [19]. Thus, we did not exclude qualitative studies from the analysis of the qualitative meta-synthesis based on their quality.

### 2.6. Characteristics of Included Studies

The research design of the included studies was as follows: nine used phenomenological methods, one used a grounded theory method, two used descriptive case studies, three used content analyses, and one used a focus group interview. 

The sample size of the studies ranged from 10 to 30, with a total of 219 nurses participating. The data collection period of each study ranged from May 2020 to November 2021, and the results of each were summarized around the main theme.

Most workplaces were nationally designated COVID-19 hospitals, but university, secondary, and tertiary hospitals were also included. The workplaces included isolation wards, infection wards, intensive care units, and screening clinics for COVID-19 patients. The Seoul metropolitan area in South Korea had the largest number of participants, and they were evenly distributed across the country, except for Gangwon Province and Jeju Island. 

The main characteristics of the studies included in this study-literature list Supplementary—as well as ‘aims of study’, and ‘the main theme identified’ are presented in Table 2.

### 2.7. Data Extraction and Synthesis

This study integrated and analyzed the experiences of nurses working in a dedicated COVID-19 hospital in Korea during the COVID-19 pandemic. The qualitative findings extracted for data synthesis were analyzed inductively using the thematic synthesis method of qualitative research developed by Thomas and Harden [20].

For this study, we used the six-step qualitative meta-synthesis procedure proposed by Noblit and Hare [21] and modified by Atkins et al. [22]. Step 1 identified nurses’ experiences during the COVID-19 pandemic as the area of interest. In step 2, we searched for and selected the literature relevant to step 1.

In step 3, we repeatedly read the selected articles for analysis within individual studies and summarized the study objectives, the main findings, and the central concepts and themes for each study.

In step 4, we continuously compared and analyzed the results of individual studies based on the tables created in steps 2 and 3, and the data, organized by study, to analyze the associations between individual studies. Analyzed.

In step 5, we identified the technical themes identified in step 4. Technical themes are the concepts, themes, and explanations presented by the authors of the analyzed papers, as well as themes based on interview quotes.

Finally, step 6 synthesizes the findings from the previous steps to explore new interpretations and theory generation. In this study, we applied a second level of synthesis to the results analyzed in step 5 to generate synthesized themes in order to draw expanded interpretations.

During the meta-synthesis process, the authors met seven times to review and discuss the results of the within-study and between-study association analyses and second-level synthesis from steps 3 through 6, coordinate their opinions, and make iterative revisions.

Three researchers (Han, S-J., Hong, H.-J., and Shin, B.-S.) read the results of each article individually and summarized the author’s name, year of publication, purpose of the study, research method, participants, department, data collection period, type, and location of the hospital. The results are shown in Table 1.

For the meta-synthesis, each researcher repeatedly and carefully read all the content, including the title, topic, categories, and citations, and extracted and organized all the codes to be analyzed. The organized codes were repeatedly reviewed based on the commonalities, differences, and similarities of each content type, and the reviewed content was categorized into subthemes. The categorized codes were continuously reviewed against other codes and revised if necessary. Finally, themes were inductively synthesized around the final classified subthemes.

## 3. Results

The synthesis of Korean hospital nurses’ experiences of caring for patients with COVID-19 resulted in 6 themes and 13 subthemes. The six themes are as follows: “The crisis of a pandemic that suddenly appeared”, “challenges in nursing patients with infectious diseases”, “a struggle in an unknown time due to a prolonged infectious disease”, “ethical dilemmas in the face of infectious diseases”, “duality of the social support system”, and “professional growth for nurses regarding infectious diseases”. Next, each topic and subtopic are described with illustrative quotes. Each major topic and subtopic are shown in Table 3.

1. The crisis of a pandemic that suddenly appeared

Participants felt the “crisis of a pandemic that suddenly appeared” as an initial emotion when COVID-19 first appeared. This manifested as a vague fear of the virus and of the rapid spread of this uncertain infectious disease. Nurses struggled during the COVID-19 pandemic without any preparation.

(1) Vague fear about COVID-19

There was a ‘vague fear of COVID-19’ in the nursing environment that none of the participants had ever experienced before.They felt the fear of being thrown into an unknown world where they had to provide nursing care without much knowledge.

Participants described it as being like World War III, where everything felt threatening and uncertain, leading to a sense of panic in the midst of the crisis (A2, A4, A6, A7, A8, A9, A10, A11).

(2) Fear of spreading infection

Participants experienced concern and anxiety regarding the possibility of contracting COVID-19 and of spreading it to others. Fear was a physical, mental, and social stress that led to the development of a catatonic fever. They also felt socially isolated, and their daily routines were disrupted as they chose to self-isolate to avoid spreading the infection to others (A1, A2, A3, A4, A5, A7, A8, A10, A11, A16).

2. Challenges in nursing patients with infectious diseases

This aspect dealt with providing care for patients with infectious diseases, which differs from that provided through conventional nursing. In this case, the difficulties caused by the confusion generated by the response guidelines for COVID-19, an unprepared-for infectious disease, and the work overload experienced by nurses emerged.

(1) The chaos of providing nursing care for an unprepared for infectious diseas

Participants experienced much confusion in their daily tasks and had difficulty understanding and implementing the often-changing guidelines and instructions. Conflicting tasks, ambiguous instructions, and unclear standards due to the quarantine situation led to confusion. This confusion escalated and was compounded when following instructions.

Participants endured uncomfortable physical pain and discomfort from wearing personal protective equipment (PPE), and experienced poor working conditions and a lack of skilled labor (A1, A2, A4, A5, A6, A7, A8, A9, A10, A11, A13, A16).

(2) Taking on the unique and distant tasks of nursing

Participants felt overwhelmed and exhausted as they were delegated tasks outside their purview, including cleaning and disinfection, excessive demands from carers and patients (deliveries, errands), and the work of other healthcare staff (caregiving, X-rays, drawing blood). These tasks were in addition to their own nursing duties and were further complicated by minimal staffing and restricted access to outsiders for fear of infection (A2, A6, A10, A13, A16).

(3) Specialized care for people with COVID-19

The COVID-19 pandemic has changed the perception that nurses can provide special nursing care for patients. Instead, they must provide holistic nursing care, which may differ from traditional nursing care. Unlike the previous task-oriented nursing care, nurses were closer to patients’ lives and practiced patient-centered nursing care. Participants talked about their daily lives with the patients and played games with them, viewing these as moments in which to fight and beat the disease together (A1, A3, A7, A8, A11, A12).

3. A struggle in an unknown time due to a prolonged infectious disease

This theme is a story of professionalism in the time of uncertainty that still remains.

(1) Burnout in sacrifice

Behind the scenes, participants who cared for patients with COVID-19 were experiencing sacrificial burnout. Despite the sacrifices made by their families and the high intensity of their work as nurses, they felt relatively deprived due to inadequate treatment (A2, A3, A4, A8, A9, A16).

(2) Having a sense of professional mission as a nurse

Participants felt a sense of pride and reward in their professional mission as nurses during COVID-19 that they had not yet felt before and considered it a valuable experience. The pride and sense of a professional mission, of being on the frontline during the pandemic, increased their motivation and self-esteem regarding nursing. Here, participants found the support of patients, family, and friends very rewarding (A2, A3, A4, A5, A6, A7, A8, A9, A10, A11, A12, A16).

4. Ethical dilemmas in the face of infectious diseases

Participants experienced ethical conflicts at both the personal and client levels during the COVID-19 pandemic. At the personal level, nurses were constrained in their behavior by their obligation to care for patients with a severe infectious disease despite the risk of exposure to infection, and by their family members due to their contact with patients with the virus. They also experienced strong ethical conflicts regarding their patients, especially feelings of grief and helplessness about not being able to ensure the dignified death of their charges.

(1) Agony over lost human dignity

Participants experienced guilt and distress due to their inability to protect human freedom and dignity during the COVID-19 pandemic, including end-of-life care and CCTV installation. They had to control their caregivers to prevent the spread of infection before a patient’s end-of-life. They could also not provide proper aftercare because they were at risk of becoming a source of infection, preventing them from enabling a patient to die with dignity. They experienced a sense of helplessness in their professional role as they were unable to deliver the deceased patient’s belongings to their families because they were infected. They also experienced ethical conflicts such as forced hospitalization and isolation against the free will of patients or their guardians, and the observation of patients through CCTV, which invaded their privacy (A2, A3, A6, A8, A9, A11, A13).

(2) Conflict between nursing principles and uncontrollable situations in infectious disease situations

The time of COVID-19 was chaotic, and nurses were challenged by the frequent changes in the beliefs regarding infection and conflict between patients and careers who were unable to accept these challenges (A9, A15, A16).

5. Duality of the social support system

This theme embodies a social campaign to thank nurses for protecting the public’s health in the difficult period of COVID-19. However, it also had another meaning, in which nurses were stigmatized as “infected” and isolated based on the perception that they were in direct contact with patients who were infected, and thus at high risk of infection themselves. It was also a collaboration between colleagues who supported each other and held the frontline of COVID-19 under difficult circumstances.

(1) Heroes in the media and stigma in the real world

While the media promoted nurses caring for patients with COVID-19 as heroes, participants who encountered those who were infected were stigmatized as carriers of infection, leading to avoidance and social isolation. Participants’ personal lives were sacrificed when they became infected, as they had to self-isolate and stay in confined spaces. They also experienced the duality of nurses being forced to make social sacrifices while hidden behind the image of being a “hero” (A1, A4, A6, A9, A14).

(2) Community of practice through peer-to-peer collaboration

As difficult as the COVID-19 pandemic has been, participants have found solace in sharing their challenges with their colleagues and encouraging each other. In the isolation ward, which felt like an island, they helped each other with protective gear, and communication between colleagues became smoother and more collaborative. Even though they had different backgrounds and experiences, they relied on each other, sharing common goals to provide quality care and form a quarantine community (A2, A4, A5, A6, A11, A14, A16).

6. Professional growth for nurses regarding infectious diseases

Participants aspired to emulate Nightingale, a nurse on a battlefield doing her best to provide care under adverse conditions. As a nurse, she gained professional knowledge and skills in infectious diseases, improved her ability to respond to infectious diseases, and gained personal growth and a sense of responsibility and mission.

(1) Improved response to infectious diseases

Participants reported that the experience of nursing during COVID-19 provided them with practical training in infectious diseases, which enhanced their professional nursing skills in terms of preparedness and response systems for future outbreaks of new infectious diseases (A1, A5, A12, A16).

(2) Promote pride in being a nurse

Participants reported that although their nursing experience during COVID-19 was challenging, they felt proud to be able to care for patients. They also felt a great sense of accomplishment when their patients improved and were discharged, safe from death’s door. These experiences made them feel rewarded by the profession and gave them a sense of responsibility and calling. It was also an opportunity to gain social recognition, and they experienced pride and growth as nurses (A2, A3, A5, A6, A7, A9, A11, A12, A15, A16).

## 4. Discussion

### 4.1. Summary

This study is a meta-synthesis of existing qualitative research on Korean hospital nurses’ experiences of caring for patients with COVID-19. Based on these findings, we suggest the need for a practical response strategy for the emergence of new infectious diseases in the future and suggest ways to improve the response. This study also provides directions for future research to better understand nurses’ experiences with COVID-19 and to develop practical interventions for hospital nurses caring for patients with emerging infectious diseases.

### 4.2. Comparisons with Existing Knowledge and Implications for Practice

Since the 2000s, the healthcare community has experienced numerous infectious diseases of national disaster proportions, including Severe Acute Respiratory Syndrome (SARS) in 2003, Influenza A virus subtype H1N1 in 2009, and MERS in 2015. This study aims to compare the holistic nursing experience of these infectious diseases and that of COVID-19.

The first theme, “the crisis of a pandemic that suddenly appeared”, was the vague fear of uncertainty and of spreading infection participants experienced while caring for patients with COVID-19. This was consistent with previous studies of nurses’ experiences with a similar infectious disease, MERS, which found that anxiety and fear were heightened by concerns regarding contagion and uncertainty about how the situation would evolve in the future [23,24]. Studies have also found that the threat of the COVID-19 pandemic was represented as a war, and that the world shared a climate of fear [25]. As nurses were in the closest contact with patients during the COVID-19 pandemic, they experienced anxiety about the possibility of infection and the transmission thereof to their families [26,27]. Thus, it is necessary to monitor their mental and psychological state and provide appropriate emotional support. In a study of SARS, an infectious disease similar to COVID-19, nurses’ negative emotions decreased and their positive attitudes increased as their knowledge of the disease increased, reducing anxiety and stress [28].

Therefore, this suggests that it is necessary to secure specialized nursing personnel who can provide sufficient education and accurate information and respond quickly, and to have a nursing system that can support a multifaceted support system.

The second theme, “challenges in nursing patients with infectious diseases” represents the confusion in infectious disease care and nurses’ overwhelming workload, caused by a lack of guidance on how to respond to the virus. Inadequate facilities, ambiguous standards, and insufficient training added to the confusion, while the physical discomfort of wearing protective equipment and the intensive workload faced by nurses led to victimization and burnout. Although the COVID-19 pandemic is different due to its prolonged duration, Korea developed guidelines and improved its epidemic prevention system in response to the risk of the transmission of a new infectious disease following the 2015 MERS outbreak [29]. Nevertheless, inadequate standards and guidelines for infectious diseases added to the confusion. Consistent with our study, research on Taiwanese nurses during an outbreak of SARS abroad found that they were confused by the lack of information, lacked adequate support (PPE, staffing), and experienced occupational stress due to a heavy workload and the operation of numerous types of equipment during nursing care [30].

Therefore, based on the experience of COVID-19, it is necessary to improve the ability to cope with future epidemics and establish a clear reference point and system to improve the quality of nursing care. An empirical study on the work of nurses in hospitals dedicated to COVID-19 showed that the principles, methods, and effects of staffing support need to be evaluated in the future, and that staffing guidelines and systems that can be used to determine appropriate assignments, numbers, and timing, considering the content and difficulty of tasks, must be established [31]. Therefore, the government and domestic medical institutions should prepare standardized guidelines regarding nursing patients with infectious diseases in advance. Furthermore, additional support such as regular infectious disease education and settling into the work environment should be provided so that medical staff can perform their tasks according to the guidelines in the event of an infectious disease outbreak [32].

On the other hand, nursing experiences during COVID-19 changed the perception of traditional nursing work. Viewing patients as human beings and interacting with them reaffirmed the value and meaning of nursing in providing holistic care for patients with COVID-19. This is similar to previous studies that showed that the pandemic led to authentic nursing [9], and that such experiences enhance the responsibility and motivation for patient care [33]. Therefore, it is necessary to promote positive emotions and adaptation so that healthcare workers can fulfill their responsibilities while being recognized as valuable human resources in a national disaster situation.

The third theme, “a struggle in an unknown time due to a prolonged infectious disease”, dealt with nurses enduring a difficult time with a sense of their mission but while experiencing burnout due to work overload in the real world. Nurses’ work overload refers to the demands of multiple conflicting roles, and the conflict between roles is known to exacerbate emotional burnout [34]. In addition, policies that minimize social contact with patients impose an additional workload on nurses, leading to burnout [35].

Healthcare systems will need to develop strategies to create a work environment that allows nurses to focus on caring for patients and to establish a clear scope of practice. In addition, to prevent burnout in the nursing workforce, nurses’ dedication should be recognized and adequately rewarded. Furthermore, the government should provide comprehensive policy support to address the wage gap between nurses, as the unequal economic rewards and inadequate treatment despite the high intensity of their work can lead to conflict among members.

In this study, nurses had a sense of professional mission during the COVID-19 pandemic, as reported in previous studies. They felt pride in providing care and fulfilling their responsibilities during a national disaster [36]. This is consistent with the pride of nurses who cared for patients with COVID-19 in Wuhan, China [37]. Support from patients, family, and friends was a source of strength and motivation, allowing nurses to endure difficult situations with a sense of a professional mission and belief that they could do this because they were nurses. Given the findings that nursing care is driven by a sense of mission [38], it is necessary to create a positive nursing environment by recognizing the importance of social support systems in overcoming infectious diseases.

The fourth theme, “ethical dilemmas in the face of infectious diseases”, deals with the multiple ethical conflicts participants faced in the chaotic healthcare setting of COVID-19. Their personal ethical values clashed with their expected roles as professional nurses in the face of an infectious disease. We explore these situations according to the four principles of ethics outlined in the 1979 Belmont Report. First, the dilemma regarding the principle of autonomy is that patients infected with COVID-19 were forcibly admitted and isolated by the government, regardless of their autonomy. Restraints were applied in uncontrollable situations, their guardians could not be with them, the doors to their rooms were locked from the outside, and there was no guarantee of privacy as patients were monitored through CCTV. Second, the dilemma of the principle of doing good is that the COVID-19 medical field did not have proper guidelines and the policies changed daily, causing inconvenience to patients who were not infected. Third, the dilemma of the principle of non-maleficence is that nurses became infected and self-isolated, leaving them unable to provide adequate care to patients due to staff shortages. Finally, the dilemma of the principle of justice was that participants were led to discriminate against the infected, as they were concerned about becoming carriers of the infection and causing harm to their families by providing nursing care for COVID-19 patients. These results were consistent with the findings of Park’s [39], who found that the principles of autonomy and of doing good were significantly influenced by the principle of justice. In Torda’s [40] study on SARS, healthcare workers were found to provide care despite the infringements on personal freedom, difficulties in maintaining privacy, and personal risk to them. Sperling’s [41] found that nurses involved in the COVID-19 response were more influenced by clinical ethics, which focuses on the values and rights of the individual client, than public health ethics, which is based on utilitarianism and focuses on the population and public good.

The fifth theme, “duality of the social support system”, is consistent with the findings of this study, as nurses’ experiences of caring for MERS patients showed they were performing important roles and functions in society as a profession. However, they also perceived themselves as victims of infection and risk factors for disease transmission [42]. In the COVID-19 pandemic, which was new to everyone, participants realized the importance of sharing knowledge and collaborating with their colleagues [43]. They described this as “comradeship” [44] and became “comrades”, sharing a sense of closeness and identification [45]. According to Chiang et al. [46], nurses who cared for patients with SARS expressed the bond between colleagues as stemming from their “being in the same boat”. This positively impacted the care provided for patients with SARS due to the improved collaboration between colleagues, which is consistent with the results of this study. In addition, the experience of patients with MERS also reflected the successful collaboration between colleagues [44,45]. In the COVID-19 pandemic, nurses’ solidarity with their colleagues was found to be a complementary positive relationship, consistent with the results of this study [47]. For nurses, the interaction between colleagues who shared the difficulties and problems in caring for patients and co-operation with each other increased their work efficiency and improved the quality of care, which positively affected patient health outcomes and made them feel more confident in their work [48].

Finally, the sixth theme, “professional growth as a nurse regarding infectious diseases”, was consistent with previous research on the MERS epidemic, which found that participants expanded their awareness of nursing by working on a new infectious disease outside their normal duties as nurses [24]. In addition, participants experienced professional growth as nurses in infectious diseases through their COVID-19 nursing experience. These findings were consistent with previous research on the MERS outbreak, which found that nurses realized their previously unrecognized roles as nurses and vowed to engage with patients with infectious diseases in the future [24,49].

In conclusion, Korean hospital nurses’ experiences of COVID-19 were consistent with the concept of resilience, which is the ability to grow and develop through the adversity arising from a new infectious disease. Resilience is a personal characteristic that enables a person to cope effectively with adversity, providing them with the positive power and strength to overcome difficulties or stressful situations, resulting in positive outcomes [50]. Nurses with such resilience can overcome the difficulties and stresses experienced in their work and enhance their professional self-identity and competence through their ability to adapt quickly to difficult tasks [51].

Polk’s [52] mid-range theory of resilience consists of a temperamental pattern, which is an individual’s internal characteristics; a relational pattern, which is about social connections and trusting relationships; a situational pattern, which is the interaction between internal characteristics and external factors to mediate risk factors; and a philosophical pattern, which includes an individual’s beliefs and life values. Of the four patterns that make up the theory of resilience, the nurses in this study experienced COVID-19 in a relational pattern, meaning that they coped with the pandemic through their relationships with family, friends, and coworkers.

Support from family, friends, and significant others increased nurses’ psychological resilience during the pandemic [53]. Support from fellow nurses enhanced their coping skills [54]. For nurses, peer support can help them handle their work more effectively and deal with difficult situations [55,56]. These experiences lead to personal growth and increased confidence in one’s own resilience [57]. Thus, resilience is the process of emerging from the life challenges that arise in a crisis, transforming the external environment into a protective environment through potential and positive forces and converting these challenges into opportunities for growth [58]. Nurses have also found that recognition of their ability to respond to infectious diseases and pride in their role has been a source of resilience [59] and enhanced their professional competence [60], and the unique experience of the COVID-19 pandemic has led to greater resilience in well-resourced and well-organized health systems [61].

### 4.3. Strengths and Limitations

This study applied Lincoln and Guba’s [62] evaluation criteria to identify the strengths and limitations of the meta-synthesis.

To establish reliability, we used a comprehensive systematic review methodology and transferred it to Endnote20 (Clarivate Analytics, Philadelphia, PA, USA) to remove duplicates. We searched, extracted, and analyzed qualitative data from various databases to maintain the reliability of our findings. We also used the ENTREQ checklist [16] to increase transparency during the review. However, the applicability may be limited by several factors. First, the nurses in this study experienced COVID-19 in a variety of healthcare settings, including dedicated COVID-19 wards, emergency departments, intensive care units, and screening clinics, and their experiences may vary depending on their practice. Second, the heterogeneity of data on Korean nurses’ experiences with COVID-19 may limit our ability to understand the experiences of the pandemic in other countries. It is also important to note that the subjective nature of the analysis makes it difficult to generalize, the analysis may be at risk of bias, and the analysis may not be representative of all nurses.

To ensure reliability, we examined and agreed on the reliability of the findings based on the original texts that were included. A logical thematic analysis process was used, and the research process was documented. In addition, three experienced qualitative researchers participated in the analysis process and analyzed the data inductively using the thematic synthesis method to generate overarching themes, subthemes, examples, and quotes through a consensus process.

The strengths of this study include the use of the CASP checklist to critically appraise the included studies and the absence of potential bias, as 5 of the 16 studies did not disclose the relationship between researchers and participants. However, there may be publication bias in this meta-synthesis, as we only included studies published in Korean and studies published in English on Korean nurses.

### 4.4. Future Research

As a qualitative meta-synthesis study of Korean nurses’ experiences of the COVID-19 pandemic, this study was able to provide an in-depth understanding of the Korean context. However, future studies need to identify and generalize the comprehensive perspectives of nurses who have experienced infectious diseases abroad.

As it was found that the drive to overcome the COVID-19 pandemic was nurtured through relational interactions, it is suggested that future research develops programs related to overcoming difficulties in nurses’ response to the pandemic.

There is also a need for comparative analytical studies to identify differences in healthcare organizations that experienced national-disaster-level infectious diseases, including COVID-19, and the perceptions and experiences of each discipline in the nursing workforce.

## 5. Conclusions

This study synthesized the COVID-19 nursing experiences of Korean hospital nurses in the context of their developmental process, and found that there were problems, such as confusion regarding work identity, ethical dilemmas, and the duality of nursing support systems. Nevertheless, the nursing experience of interacting with patients, the professional mission and commitment of nurses, and the deep intimacy of colleagues working together enabled them to overcome the challenges presented by the pandemic in a positive way.

## Figures and Tables

**Table 1:** Quality appraisal of included studies.

No.	1	2	3	4	5	6	7	8	9	10
	Clear Statement of Aims	Qualitative Methodology	Research Design	Sampling	Data Collection	Reflexivity	Ethical Issues	Data Analysis	Findings	Value of Research
A1	Y	Y	Y	Y	Y	Y	Y	Y	Y	Y
A2	Y	Y	Y	Y	Y	N	Y	Y	Y	Y
A3	Y	Y	Y	Y	Y	Y	Y	Y	Y	Y
A4	Y	Y	Y	Y	Y	N	N	Y	Y	Y
A5	Y	Y	Y	Y	Y	Y	Y	Y	Y	Y
A6	Y	Y	Y	Y	Y	N	Y	Y	Y	Y
A7	Y	Y	Y	Y	Y	Y	Y	Y	Y	Y
A8	Y	Y	Y	Y	Y	Y	Y	Y	Y	Y
A9	Y	Y	Y	Y	Y	Y	Y	Y	Y	Y
A10	Y	Y	Y	Y	Y	Y	Y	Y	Y	Y
A11	Y	Y	Y	Y	Y	Y	Y	Y	Y	Y
A12	Y	Y	Y	Y	Y	Y	Y	Y	Y	Y
A13	Y	Y	Y	Y	Y	N	Y	Y	Y	Y
A14	Y	Y	Y	Y	Y	Y	Y	Y	Y	Y
A15	Y	Y	Y	Y	Y	Y	Y	Y	Y	Y
A16	Y	Y	Y	Y	Y	N	Y	Y	Y	Y

Agreed assessment for each study: Yes (Y)/No (N).

**Table 2:** Overview of studies included in the meta-synthesis (N = 219).

Author (Year)	Study Aim	Methodology	Participants Working Place	Time Period	Type of Hospital	Location	Main Theme Identified/Result
Jin, D. L., and Lee, G. Y. 2020 [A1]	To understand the experiences of nurses working in a situation where the entire hospital is temporarily closed except for inpatient care due to an outbreak of COVID-19 in the hospital.	phenomenological method Colaizzi’s	10 nursing care integration ward, outpatient safety clinic center, isolation ward, screening clinic center, surgical intensive care unit etc	1 May 2020– 13 June 2020	University hospital	Seoul	COVID-19 came without preparation. Nursing work and sense of vocation. Life changed by COVID-19.
Kim, M. Y,. Kim, S. S., and Sim, J. E. 2023 [A2]	To explore the essential structure and meaning of the in-depth experience of nurses caring for COVID-19 patients.	phenomenological method Colaizzi’s	10 COVID-19 patients’ isolation ward	4 June 2020– 23 June 2020	General hospital	Seoul	The prelude to war. Surviving the war. Unfinished battle. Flowers that bloom in the battlefield.
Kim N. H,. Yang, Y. R., and Ahn, J. H. 2022 [A3]	To understand the experiences of nurses caring for COVID-19 patients in infection wards dedicated to COVID-19 through an in-depth exploration of the patient experience and meaning of care.	content analysis	14 COVID-19 patients’ isolation ward	4 July 2020– 30 August 2020	National COVID-19 designated hospitals	Jeollabukdo Province	Struggling to prepare an infection ward. Fear and anxiety about infection. The weight of pressure from patient care. Efforts to protect patients. Maturity of professional identify as a nurse. A quarantine community that we create together.
Oh, H. L., and Lee N.K. 2021 [A4]	To explore the deep structure and meaning of nurses’ experiences of caring for patients with COVID-19.	phenomenological method Colaizzi’s	16 General ward, Intensive care unit, screening center, isolation ward	30 June 2020– 30 September 2020	National COVID-19 designated hospitals	Gyeonggi, Gyeongbuk, Daegu, Jeonnam	A repetitive sense of crisis. Enduring a drastic change. Sacrifice of personal life. Pride in nursing.
Lee, H. J,. et al., 2022 [A5]	To explore the motivation and lived experience of frontline nurses responding to the COVID-19 pandemic in South Korea.	phenomenological method Colaizzi’s	10 general ward, intensive care units	August 2020– November 2020	National COVID-19 designated hospitals	Seoul	Decision to participate in the COVID-19 response. Facing hardship. Distress due to the nature of COVID-19. Overcoming hardship. Growing through the COVID-19 response. The need for reciprocity.
Jang, H. Y., Yang, J. E., and Shin, Y. S. 2022 [A6]	To investigate the meaning and essence of nurses’ experiences of caring for patients with COVID-19 using a phenomenological research method.	phenomenological method Colaizzi’s	14 COVID-19 Isolation ward	20 October 2020– 15 January 2021	COVID-19 infectious disease hospital	Seoul and Gyeonggi Province	Nurses struggling under the weight of dealing with an infectious disease. Challenges added to difficult caring. Double suffering from patient care. Support for caring. Expectations for post-COVID-19 life
Lee, J. Y,. et al., 2022 [A7]	To explore the experiences of frontline nurses who provided care for patients with COVID-19 in South Korea.	descriptive qualitative study	14 general ward, infection ward, emergency room, intensive care units	December 2020– April 2021	The secondary & the tertiary general hospitals	9 hospitals in 5 provinces in South Korea	Feeling forced into a world of uncertainty. Providing unique care for COVID-19 patients. Perceiving barriers to providing quality care. Seeking meaning in caring for COVID-19 patients.
Lee, J. H., and Song, Y. S. 2021 [A8]	To develop a situation-specific theory to explain nurses’ experiences of the COVID-19 crisis.	Corbin and Strauss’s grounded theory method	16 emergency isolation ward, isolation ward, intensive care unit	2 September 2020– 20 January 2021	The tertiary general hospitals	Daegu	The chaos of being exposed defenselessly to an unexpected pandemic. Fear caused by a nursing care field reminiscent of a battlefield. Moral distress from failing to protect patients’ human dignity. Feeling like the scapegoat of the hospital organization. Increasing uncertainty due to the unpredictable state of COVID-19 Relative deprivation due to inappropriate treatment Suffering alone while experiencing the dedication of the COVID-19 hero image Gratitude for those who care for broken hearts Getting used to repetitive work. Efforts to find a breakthrough. Take heart Becoming an independent nurse. Frustration with the unchanging reality
Chung, S. J., Seong, M. H., and Park, J. Y. 2022 [A9]	To explore nurses’ experiences caring for patients with COVID-19 and examine factors that influence this in the domestic context.	conventional content analysis	10 COVID-19 isolation ward	13 January 2021– 20 January 2021	COVID-19 ward of a public hospital	Jeollabukdo Province	Unstable psychological status. Adaptation and self-esteem.
Noh, E. Y., et al., 2021 [A10]	To explore nurses’ experience with caring for patients with COVID-19 in a negative pressure room	focus group interviews	19 COVID-19 isolation ward	17 February 2021– 25 February 2021	National COVID-19 designated hospitals	Seoul	Struggling in an isolated space. Limitations of nursing infrastructure and system.
Oh, I. O., Yoon, S. J., and Nam, K. A. 2021 [A11]	To identify and describe the meaning of the work experience of nurses caring for patients with COVID-19 in a COVID-19 hotspot hospital from the perspective of the study participants.	Content analysis	30 COVID-19 isolation ward	March 2021– June 2021	National COVID-19 designated hospitals	Gyeonggido Province	Hesitating to move forward. Standing up with the name of nurse. Feeling unfamiliarity and confusion. Walking on thin ice everyday. Getting used to working. Advancing one step further. Ending up with something unsolved.
Shin, S. Y., and Yoo, H. J. 2022 [A12]	To provide an in-depth understanding of caring and communication experiences among nurses in COVID-19 units.	qualitative descriptive design	15 hematology and oncology ward, intensive care units, infection ward	July 2021– August 2021	The tertiary hospitals	Seoul	Central role of therapeutic communication. Compassion that deepens naturally. Expansion of professionalism in nursing.
Je, N. J., et al., 2022 [A13]	To provide basic data for improving the coping ability of clinical nurses in the COVID-19 pandemic.	Phenomenological method Colaizzi’s	12 artificial kidney room, neonatal intensive care unit, emergency room, COVID-19 ward	October 2021– November 2021	Hospital	Gyeongsangnamdo Province	Agony in facing a situation violating the principle of respect for man’s life and dignity. Frustration and confusion from not being able to keep the principle of doing good deeds and the prohibition of evil deeds. Doubt about fulfilling and not fulfilling the principle of justice
Park, H. J. and Choi, K. S. 2021 [A14]	To explore the meaning of the experiences of nurses working at COVID-19 drive-through screening centers	phenomenology research method	8 general ward, infection ward, screening clinic	June 2020– August 2020	Drive-through COVID-19 screening clinic	Gyeongsang bukdo	A sense of calling as a nurse. Physical and psychological stress. Daily life tailored to the work of the screening clinic. Time to live together in the fight against the virus. New perception and reward for nursing.
Ha, B.Y. et al., 2022 [A15]	To gain an in-depth understanding of the meaning and nature of the triage work experience of COVID-19 triage nurses in a general hospital.	Colaizzi (1978) phenomenological methods	14 screening clinic	11 May 2021– 20 June 2021	Screening clinics	Gyeongsang namdo 2 hospitals	Hospital superintendent on the frontline of the COVID-19 pandemic. The struggle for firewall shooters. Stuck like a Mobius belt. What keeps me going as a triage nurse.
Choi, S.Y. 2021 [A16]	To suggest complementary institutional and policy individual measures to improve the mental health of nurses in screening clinics.	phenomenological methods	7 screening clinics in emergency departments	November 2021	Screening clinics	Seoul	Performance of special types of work due to COVID-19. Lack of a support system. Inefficient guidelines. Anxiety regarding infection and transmission has dulled. Power to continue working.

A1~A16 listed in Supplementary.

**Table 3:** Identified overarching themes and domain-specific technical topics.

Major Theme	Sub-Theme	Distribution of the Main Theme	Illustrative Quotes
1. The crisis of a pandemic that suddenly appeared	(1) Vague fear of COVID-19	(A2, A4, A6, A7, A8, A9, A10, A11)	*“I’ve never seen anything like this in all my years as a nurse, not even MERS, and it’s really scary. I’m scared because I’m afraid that the patient who greeted me this morning will suddenly not be able to breathe at lunch, that they’ll suddenly get worse, and I won’t be able to help them. I’ve never seen anything like this before, a virus this big. It’s scary. It’s like a war. It’s like we’re in this situation”. Page 8 (A2)*
	(2) Fear of spreading infection	(A1, A2, A3, A4, A5, A7, A8, A10, A11, A14, A15, A16)	*“I think I was very worried about infection because the moment I got it, the people that I came in contact with, my family, plus my staff, literally the whole hospital would have to go through it. I think I was only worried about one thing; I think I put everything else aside and I was only worried about infecting other people and having other people suffer”. Page 661 (A11)*
2. Challenges in nursing patients with infectious diseases	(1) The chaos of unprepared infectious disease nursing	(A1, A2, A4, A5, A6, A7, A8, A9, A10, A11, A13, A14, A16)	*“Due to the nature of the shifts, understanding and executing the same instructions is different for everyone. There are a lot of instructions that are given that are too exceptional or vague in their criteria. It varies too much from situation to situation”. Page 416 (A1)*
	(2) Taking on the unique and distant tasks of nursing	(A2, A6, A10, A13, A16)	*“Above all, the most challenging thing is the social perspective of ‘these people are working in an isolation hospital now.’ People close to me have this kind of perspective… When?* *one of the nurses is reported on the news or the media as a confirmed patient, we also feel like cringing. Such social perspectives were very hard for us because we’ve become people that the public wants to avoid, rather than them feeling appreciation for us and thinking of us like we are working hard and trying our best”. Page 8 (A6)*
	(3) Specialized care for people with COVID-19	(A1, A3, A7, A8, A11, A12)	*“I really cared for ‘everything’ about these patients, from head to toe. Nursing is providing what the patient needs the most. In this aspect, nurses are the only people they can rely on in the isolation ward and we had to take care of everything”. Page 54 (A7)*
3. A struggle in an unknown time due to a prolonged infectious disease	(1) Burnout in sacrifice	(A2, A3, A4, A8, A9, A16)	*“We were told that they don’t pay us, so we thought that they don’t pay us, and we were okay with that, but the teachers (from outside the hospital) were paid close to 10 million won. I was shocked and felt deprived, and after I found out, I thought it was so unfair, because we do the same work, and even more work than the teachers from outside the hospital”. Page 695 (A8)*
	(2) Having a sense of professional mission as a nurse	(A2, A3, A4, A5, A6, A7, A8, A9, A10, A11, A12, A14, A15, A16)	*“If there was a war, I think we’d be in the same position as the Nightingales would be in. We wouldn’t be carrying guns, but we’d be in the same position as them in a field hospital nursing patients. So I think it’s a war. We’re in a little war and we can go and help, and I’m proud to be a nurse”. Page 243 (A14)*
4. Ethical dilemmas in the face of infectious diseases	(1) Agony over lost human dignity	(A2, A3, A6, A8, A9, A11, A13)	*“When you die, you’re treated, you’re covered with sheets, and now the funeral director comes and does that and takes you away, and they wrap you in clear plastic because you’re an infectious patient, and then they wrap you in black plastic, and then they wrap you in black plastic, and it’s like… The sight of the patient that I see on the CCTV, the feeling of not being respected as a human being, and when I die, I will tell my family that I closed my eyes without seeing my loved ones. So it hurts me so much when I think about it now”. Page 664 (A11)*
	(2) Conflict between nursing principles and out-of-control situations during infectious disease outbreaks	(A9, A15, A16)	*“You can be admitted to the hospital if you have a timely coronavirus test result, but to be admitted to the emergency room, you need to have a coronavirus test taken the same day. Even if you take the test and get the result in the morning, you must take the test again and wait for a while because the test was taken yesterday. The emergency room is becoming overcrowded. I think the reason for the long waits is that there are so many unnecessary and inefficient procedures like that. I don’t think it reflects the conditions on the ground at all. I don’t know what the rationale is for doing that and I don’t know who it’s for”. Page 48 (A16)*
5. Duality of the social support system	(1) Heroes in the media and stigma in the real world	(A1, A4, A6, A9, A14).	*“Above all, the most challenging thing is the social perspective of ‘these people are working in an isolation hospital now.’ People close to me have this kind of perspective… When one of the nurses is reported on the news or the media as a confirmed patient, we also feel like cringing. Such social perspectives were very hard for us because we’ve become people that the public wants to avoid, rather than them feeling appreciation for us and thinking of us like we are working hard and trying our best. “ Page 8 (A6)*
	(2) Community of practice through peer-to-peer collaboration	(A2, A4, A5, A6, A11, A14, A16).	*“To be honest, I think I’m able to endure hard times thanks to my companionship. It’s hard for us all. And fortunately, all colleagues are friendly, and many are so considerate of each other. We’re not pushing each other to go in, but we are voluntarily working. Even though COVID-19 is hard for me, this companionship has helped me learn and endure with them until now. “ Page 8 (A6)*
6. Professional growth of nurses regarding infectious diseases	(1) Improve response to infectious diseases	(A1, A5, A12, A16)	*“I’ve only had training and I’ve never really used PPE [personal protective equipment] and I’ve never used a negative pressure tent or a negative pressure wheelchair before COVID, so I was scared that I wouldn’t be able to do it because of the training. But when I actually tried it on and did it, I got it, and now I have the confidence to do it. I think that’s one of the things I’ve gained from working here”. Page 55 (A16)*
	(2) Promote pride in being a nurse	(A2, A3, A5, A6, A7, A9, A11, A12, A15, A16)	*“I’m a nurse, I should help. It’s something that I’m supposed to do. It’s something that I can do in a way because I’m a nurse. (Interruption) It was rewarding, it was fun, and if there’s a place that wants to send me somewhere like this again, I want to go”. Page 116 (A3)*

## Data Availability

The datasets generated during and/or analyzed during the current study are available from the corresponding author on reasonable request.

## Supplementary Materials

The following supporting information can be downloaded at: https://www.mdpi.com/article/10.3390/healthcare12090903/s1.

## Appendix A. Search Strategy to Identify Relevant Trials from PubMed

**Search**	**Query**	**Items Found**
#1 MeSH #2 Natural Term	“Nurses” [MeSH Terms]	99,829
	“nurse” [Title/Abstract]	331,507
#3	#1 OR #2	373,814
#4 MeSH	“COVID-19” [MeSH Terms]	252,446
#5 Natural Term	“experience” [Title/Abstract]	852,298
#6 MeSH	“korea” [MeSH Terms]	59,960
#7	#3 AND #4 AND #5 AND #6	6
