# Supplementary material for: Korean Hospital Nurses’ Experiences with COVID-19: A Meta-Synthesis of Qualitative Findings
Source: Healthcare (Basel). 2024 Apr 26;12(9):903. doi: 10.3390/healthcare12090903 (PMC11083708; doi:10.3390/healthcare12090903)
Supplement: Supplementary file 1 [file healthcare-12-00903-s001.zip › healthcare-2893596-supplementary.pdf]

List of Articles Analyzed

A1. Jin, D.R.; Lee, G.Y. Experiences of nurses at a general hospital in Seoul which is temporarily closed due to COVID-19. *J. Korean Acad. Soc. Nurs. Educ*. **2020**, 26, 412–422.doi:10.5977/jkasne.2020.26.4.412.

A2. Kim, M.Y.; Kim, S.S.; Sim, J.E. Overcoming experience of nurses caring for patients with COVID-19 in a hospital in Seoul. *J Korean Acad Nurs*. **2023**, 7, 1, 77-87. Doi:10.34089/jknr.2023.7.1.77.

A3. Kim, N.H.; Yang, Y.R.; Ahn, J.H. Nurses' experiences of care for patients in coronavirus disease 2019 infection wards during the early stages of the pandemic. *Korean J. Adult Nurs*. **2022**, 34, 109–121. doi:10.7475/kjan.2022.34.1.109.

A4. Oh, H.; Lee, N.K. A Phenomenological Study of the Lived Experience of Nurses Caring for Patients with COVID-19 in Korea. *J. Korean Acad. Nurs*. **2021**, 51, 561–572. Doi:10.4040/jkan.21112.

A5. Lee, H. J.; Lee, S. E.; Sang, S.; Morese, B. The lived experience of nurses who volunteered to combat the COVID-19 pandemic in South Korea: A qualitative phenomenological study. *J. Nurs. Manag*. **2002**, 30, 4, 864-871. Doi:10.1111/jonm.13571.

A6. Jang, H.-Y.; Yang, J.-E.; Shin, Y.-S. A Phenomenological Study of Nurses' Experience in Caring for COVID-19 Patients. *Int. J. Environ. Res. Public Health*. **2022**, 19(5), 2924. https://doi.org/10.3390/ijerph19052924

A7. Lee, J. Y.; Lee, S.; Choi, H.; Oh, E. G. Exploring the experiences of frontline nurses caring for COVID-19 patients. *Int. Nurs. Rev*. **2022**, 70(1), 50–58. https://doi.org/10.1111/inr.12801.

A8. Lee, J.H.; Song, Y. Nurses' Experiences of the COVID-19 Crisis. *J. Korean Acad. Nurs*. **2021**, 51(6). https://doi.org/10.4040/jkan.21160.

A9. Chung, S.; Seong, M.; Park, J. Nurses' Experience in COVID-19 Patient Care. *J. Korean Acad. Nurs. Adm*. **2022**, 28(2), 142–153. https://doi.org/10.11111/jkana.2022.28.2.142

A10. Noh, E.-Y.; Chai, Y.J.; Kim, H. J.; Kim, E.; Park, Y.-H. Nurses' Experience with Caring for COVID-19 Patients in a Negative Pressure Room Amid the Pandemic Situation. *J. Korean Acad. Nurs*. **2021**, 51(5), 585–596. https://doi.org/10.4040/jkan.21148

A11. Oh, I. O.; Yoon, S. J.; Nam, K. A. Working Experience of Nurses at a COVID-19 Dedicated Hospital. *Korean J. Adult Nurs*. **2021**, *33*(6), 657–669. https://doi.org/10.7475/kjan.2021.33.6.657

A12. Shin, S.; Yoo, H.J. Frontline nurses' caring experiences in COVID-19 units: A qualitative study. *J. Nurs. Manage*. **2022**, ;30(5), 1087–1095. https://doi.org/10.1111/jonm.13607.

A13. Je, N.J.; Lee, D.-Y.; Kwon, M.K.; Yoo,J.-E. A-Young Jo. Experience of Clinical Nurse's Bioethics Dilemma during the COVID-19. *J. Korea Convergence Society*. 2022, 13(3), 379-390. https://doi.org/10.15207/JKCS.2022.13.03.379

A14. Park, J.P.; Choi, K.S. Experience of Nurses Working at the Drive-Thru COVID-19 Screening Clinic. *J Korean Acad Nurs Adm. **2021**,* 27(4), 236–247. Doi: 10.11111/jkana.2021.27.4.236

A15. Ha, B.Y.; Bae, Y.S.; Ryu, H.S.; Jeon, M.K. Experience of Nurses in Charge of COVID-19 Screening at General Hospitals in Korea. *J Korean Acad Nurs Adm.* **2022**. 52(1), 66–79. Doi:10.4040/jkan.21166

A16. Choi, S.Y. Job stress experiences caused by COVID-19 related work of screening center nurses: For a tertiary hospital in Seoul. Master's Thesis, Yonsei University, Seoul, Republic of Korea, December 2021.
